# Editors’ Book Table

**Published:** 1875-05

**Authors:** 


### Editors' Book Table.

[NOTE. — All works reviewed in the pages of the CHICAGO MEDICAL JOURNAL may be found in the extensive stock of W. B. KEEN, COOKE & Co., whose catalogue of Medical Books will be sent to any address upon request.]

DENTAL PATHOLOGY AND SURGERY. By *S. James A. Salter, M.B., F.R.S.*, Member of the Royal College of Surgeons, Examiner in Dental Surgery at the College, Dental Surgeon to Guy's Hospital. New York: William Wood & Co., Publishers. 1875.

The author of this work will be recognized by surgeons as likewise author of the article on "Surgical Diseases of the Teeth," in Holmes' System of Surgery. Having had the advantage of a previous medical education, and some years subsequent practice as surgeon, he comes to his task as an instructor in the department of dental surgery better prepared than the majority of his predecessors who have enriched the literature of this specialty. The chapters upon the anatomy—normal and morbid—of the teeth are unusually full and explicit. Not only the special forms of these organs, and the deformities and irregularities to which they are liable, are elaborated carefully, but their general anatomy, together with that of their accessory structures, are carefully considered.

The subject of the pathology of the teeth is treated comprehensively and in full detail, and in the chapters devoted to the special subject of the nervous relations of the dental apparatus, a wide, and hitherto almost unexplored, field of investigation is opened up to the student. The portion of the work devoted to the subject of the mechanical appliances for the treatment of deformities, displays sound judgment and thorough appreciation of the necessities of each case. We know no better book upon the subject than this volume, and doubt if one so good is within the reach of the American student. H.

Essentials of the Principles and Practice of Medicine. A Handbook for Students and Practitioners. By *Henry Hartshorne*, *A.M.*, *M.D.*, Professor of Hygiene in the University of Pennsylvania, etc., etc. Fourth Edition. Thoroughly Revised, with One Hundred Illustrations. Philadelphia: Henry C. Lea. 1874.

That three editions of a work should have been already exhausted, and the issue of a fourth demanded in a comparatively short time, is a criticism sufficiently favorable to satisfy the self-love of any author. Nor will we attempt to detract from its force by any unfavorable comments upon one of the best books of its kind extant. Upon general principles, we cannot accord a hearty approval to compendiums as a class, believing that, while they will subserve for some their legitimate purpose, for the majority they will be made pretexts for sciolism. The work is divided into two parts, the first containing four sections, the first of which comprises an excellent summary of general pathology as presented in the morbid states of the system at large and of special organs, concluding with a brief statement of the different modes of death. The second section, upon semeiology, includes symptomatology, physical diagnosis, with descriptions of the operative procedures and apparatus for laryngoscopy, diagnosis of diseases of women, ophthalmoscopy, sphygmography, thermometry, and pneumatic aspiration, with directions for conducting post-mortem examinations and medico-legal investigations in cases of suspected poisoning. In regard to the mode of conducting post-mortem examinations of the spinal cord, we will venture to suggest an improvement upon the "chisel and mallet or saw," for facilitating section of the vertebral arches—a pair of strong cutting pliers, the blades bent at an obtuse angle to the handle. In section third, upon "general therapeutics," we are sorry to see the author holding on with such tenacity to the use of "blood-letting in the treatment of violent inflammations and congestions." This seems like turning the wheels of progress backwards. Part second, upon "special pathology and practice," constitutes nearly three-fourths of the bulk of the volume, to which we must object, as being entirely too extensive to be sufficiently comprehensive. H.

BOOKS RECEIVED.

TRANSACTIONS OF THE TWENTY-FOURTH ANNIVERSARY MEETING OF THE ILLINOIS STATE MEDICAL SOCIETY—Held in the City of Chicago, May 19, 20, 21, 1874.

PAMPHLETS RECEIVED.

ON CERTAIN MORBID ALTERATIONS OF MUCOUS MEMBRANE: Their Influence on Speech and Their Apparent Relations with Diseased Nerve Structure. By *Beverly Robinson, M.D.*, Surgeon to the Manhattan Eye and Ear Hospital, Department of the Throat. Formerly Resident Physician to the Paris Hospitals, etc., etc. Reprinted from the New York Medical Journal, March, 1875. New York: D. Appleton & Co. 1875.

THE NASAL DOUCHE: What it Accomplishes, and What it Does Not. By *Beverly Robinson, M.D.*

THE FOURTEENTH ANNUAL REPORT of the Trustees, Superintendent and Treasurer of the Illinois State Hospital for the Insane, Jacksonville. 1874.

VALEDICTORY ADDRESS to the Medical Graduates of the University of Louisville, March 1, 1875. By *D. W. Yandell*, Professor of the Science and Art of Surgery and Clinical Surgery.

FIFTH REPORT of the New York Ophthalmic and Aural Institute, for the Twenty Months beginning May 1, 1873, and ending December 31, 1875.

CLINICAL STUDIES, with Large Emetic Doses of Ipecacuanha. Reprinted from the Atlanta Medical and Surgical Journal.

ON THE HOMŒOPATHICITY OF ELECTRICITY: Where Indicated, and where Electricity may be Potentized. By *R. N. Tooker, A.M., M.D.*, Chicago. 1875.

A CASE OF REFLEX NEURALGIA, Associated with Urethral Contractions, and a Rare Form of Urinary Sinus, with a Description of the Cold Water Coil. By *Fessenden N. Otis*, Clinical Professor of Genito-Urinary Diseases, College of Physicians and Surgeons, New York. Reprinted from the New York Medical Journal, Feb., 1875.

CHOLERA AND PLANETARY EPIDEMICS. By *Richard Mansell*, Rock Island, Ill. 1875.

JOURNALS RECEIVED.

L'Anatomie et de la Physiologie, etc., Journal de—Charles Robin. Paris. No. 2.
The American Medical Weekly—Vol. ii, Nos. 14, 15, 16.
The American Practitioner—Vol. xl, No. 64.
The American Journal of the Medical Sciences—No. cxxxviii.
The Atlanta Medical and Surgical Journal—Vol. xiii, No. 1.
The American Journal of Insanity—Vol. xxxi, No. 4.
The Clinic—Vol. viii, Nos. 13, 14, 15, 16.
The Cincinnati Lancet and Observer—Vol. xviii, No. 4.
The Detroit Review of Medicine and Pharmacy—Vol. x, No. 4.
The Dental Cosmos—Vol. xvii, No. 4.
The Eclectic Medical Journal—Vol. xxxv, No. 4.
La Gazette Medicale de Paris—Tome iv, No. 10.
The Indiana Journal of Medicine—Vol. v, No. 12.
The Kansas City Medical Journal—Vol. v, No. 2.
The London Lancet, March, 1875.
The Laboratory, Boston—Vol. i, No. 9.
The Medical Register and Advertiser—Vol. i, No. 1.
The Medical & Surgical Reporter—Vol. xxxii, Nos. 13, 14, 15,16.
The Medical Record—Vol. x, Nos. 13, 14, 15, 16.
The Medical Times (Philad.)—Vol. v, Nos. 178, 179, 180, 181.
The Medical News and Library—No. 388.
The Monthly Abstract of Medical Science—Vol. ii, No. 4.
The Medical Examiner—Vol. xvi, No. 7.
The Medical Herald, Leavenworth—Vol. viii, No. 10.
The Nashville Journal of Medicine and Surgery—Vol. xv, No. 4.
The New York Medical Journal—Vol. xxi, No. 4.
The New Remedies—Vol. iv, No. 2.
The Practitioner, London—No. lxxxi.
The Pharmacist, Chicago—Vol. viii, No. 4.
The Pharmacal Gazette—Vol. iii, Nos. 64, 65.
The Peninsular Journal—Vol. ii, No. 4.
El Repertorio Jalisciense—No. 9, Tomo i.
The Richmond and Louisville Medical and Surgical Journal—Vol xix, No. 4.
The St. Louis Clinical Record—Vol. ii, No. 1.
The St. Louis Medical and Surgical Journal—Vol. xii, No. 8.
The Southern Medical Record—Vol. v, No. 2.
The United States Medical Investigator—Vol. i, No. 7.
The Virginia Medical Monthly—Vol. ii, No. 1.

